# Revealing Goal-Directed Neural Control of the Pharyngeal Phase of Swallowing

**DOI:** 10.1007/s00455-024-10758-3

**Published:** 2024-10-10

**Authors:** Shahryar Zainaee, Brent Archer, Ronald Scherer, Verner Bingman, Mehran Ghasemi

**Affiliations:** 1 Department of Communication Sciences and Disorders, College of Health and Human Services, Bowling Green State University, Bowling Green, OH USA; 2 Department of Psychology, J. P. Scott Center for Neuroscience, Mind and Behavior, Bowling Green State University, Bowling Green, OH 43403 USA

**Keywords:** Dysphagia, Swallowing, Swallowing Neurology, Swallowing Physiology, Swallowing Neurophysiology, Goal-directed Swallowing, Deglutition, Deglutition Disorders

## Abstract

Swallowing is considered a three-phase mechanism involving the oral, pharyngeal, and esophageal phases. The pharyngeal phase relies on highly coordinated movements in the pharynx and larynx to move food through the aerodigestive crossing. While the brainstem has been identified as the primary control center for the pharyngeal phase of swallowing, existing evidence suggests that the higher brain regions can contribute to controlling the pharyngeal phase of swallowing to match the motor response to the current context and task at hand. This suggests that the pharyngeal phase of swallowing *cannot be exclusively reflexive or voluntary* but can be regulated *by the two neural controlling systems*,* goal-directed and non-goal-directed*. This capability allows the pharyngeal phase of swallowing to *adjust appropriately based on cognitive input*,* learned knowledge*,* and predictions*. This paper reviews existing evidence and accordingly develops a novel perspective to explain these capabilities of the pharyngeal phase of swallowing. This paper aims (1) to integrate and comprehend the neurophysiological mechanisms involved in the pharyngeal phase of swallowing, (2) to explore the reflexive (non-goal-directed) and voluntary (goal-directed) neural systems of controlling the pharyngeal phase of swallowing, (3) to provide a clinical translation regarding the pathologies of these two systems, and (4) to highlight the existing gaps in this area that require attention in future research. This paper, in particular, aims to explore the complex neurophysiology of the pharyngeal phase of swallowing, as its breakdown can lead to serious consequences such as aspiration pneumonia or death.

## Swallowing Mechanism

Swallowing is an ability that results from a set of movements that allows all of us to eat or drink. Swallowing generally works seamlessly, whether dealing with saliva or a dense food bolus regardless of one’s identity. In an evolutionary context, the crossing of the respiratory and digestive tracts has evolved into a unique aerodigestive pathway among humans [1]. This change can increase the risk of dangerous consequences, such as choking or other life-threatening situations. Therefore, this type of aerodigestive pathway has likely influenced our breathing and swallowing patterns.

The swallowing mechanism is traditionally considered a three-phase process, including oral, pharyngeal, and esophageal phases. Although various studies have attempted to illuminate the physiology and neurology of swallowing within each phase, a consistent agreement describing the neurophysiology of the pharyngeal phase of swallowing (PS) has not been well established. Indeed, remarkable explorations have been conducted to study the neurophysiology of the PS, but these investigations have not specifically examined the neurophysiology of the PS itself in isolation. As a result, the bulk of existing evidence on the physiology or neurology of the PS has been measured predominantly following the oral phase. The oral phase can be considered the voluntary stage of swallowing, which can be completely interrupted or volitionally regulated under cognitive processing [2]. The adaptable nature of the oral phase of swallowing under cognitive processing can make it a good example of a voluntary (goal-directed) behavior (see the next paragraph). This phase cannot only change based on the ongoing context and task at hand but may also affect how the PS works afterward [3]. In other words, the way the oral phase starts or progresses can impact the PS process. For example, consuming a bolus from a small cup [4] or through a straw [5] can require different levels or amount of cognitive processing and may provide different effects on the PS process [6, 7]. Therefore, differences among subjects or variations in methodological approaches, experimental designs, protocols, and analyses may cause inconsistencies across the reported evidence.

The PS can be consciously triggered, although it usually begins without conscious perception [2]. Suppressing the PS during feeding or at rest is challenging, but the oral phase can be stopped at any time [2]. These characteristics of the PS can support the idea that the nervous system may regulate the PS process through both voluntary and reflexive systems. In this regard, Francois Clarac described the term reflex as a simple and fast reaction to the environment, which is mediated at the lower motor neuron [8]. Jonathan Wolpaw further explained that behaviors are reactions to stimuli, and the distinction between voluntary and reflex behaviors also depends on the nature of the stimuli involved [8]. Wolpaw also clarified that reflexive behaviors result from unknown (unfamiliar) stimuli, but they can become predictable if the stimuli are known. The known stimuli thus lead to voluntary behaviors, but unknown stimuli typically evoke reflexive behaviors. Voluntary behaviors can be also termed goal-directed, while involuntary behaviors can be labeled non-goal-directed. Goal-directed behaviors also enable one to keep a task constantly active while monitoring the current context and filtering noises from inappropriate sources [9]. A goal-directed behavior relies on predictions about the future consequences of the motor response according to the current context and can be voluntarily intended to produce efficient responses to achieve desirable outcomes based on expectations [10, 11]. In simpler terms, one can respond more effectively and swiftly by anticipating what will occur. The sensorimotor interaction of these behaviors (goal-directed and non-goal-directed) might vary depending on one’s characteristics, experiences, type of stimuli involved, the context (environment), etc [8]. In addition, all behaviors can exist along a spectrum ranging from entirely reflexive to entirely voluntary, with none being exclusively one or the other. However, these capacities and their extent have not been thoroughly understood yet in the context of the PS.

Understanding these capacities and their extent poses significant importance regarding the PS as it lasts for about a second and involves a complex set of movements to ensure safe swallowing [12, 13]. In order to facilitate quick adaptive responses like the PS, the nervous system may need to predict the consequences of the perceived sensory information about the probable external and internal sources of sensory signals, even before or without direct access to these sources, to ensure the quickness of flawless responses [14]. These pre-existing predictions before receiving stimuli can influence or shape the efficient PS responses. Therefore, this paper aims to provide a consistent explanation (1) to integrate and comprehend the neurophysiological mechanisms involved in the PS, (2) to explore the reflexive (non-goal-directed) and voluntary (goal-directed) neural systems of controlling the PS, (3) to provide a clinical translation regarding the pathologies of these systems, and (4) to highlight the existing gaps in this area that require attention in future research. This paper, in particular, aims to explore the complex neurophysiology of the PS, as its breakdown can lead to serious consequences such as aspiration pneumonia or death.

## Non-Goal-Directed Neural Control of the Pharyngeal Phase of Swallowing

The brainstem has conventionally been considered the primary center controlling the process of the PS. In this regard, supporting evidence has led to the development of a functional model coordinated by a swallowing central pattern generator (see Table 1) (SCPG). Indeed, this model identifies the medulla oblongata in the brainstem as a central pattern generator of swallowing [15]. The SCPG can produce PS responses under the influence of prompt sensory feedback because of peripheral afferent information [16, 17].

Once a bolus reaches the pharynx, the SCPG receives sensory information through the glossopharyngeal and vagus nerves, which ascend sensory information to the nucleus tractus solitarius (NTS). Accordingly, neurons situated in the intermediate, ventromedial, and interstitial subnuclei of NTS receive the conveyed sensory information [18]. Retrograde labeling studies have revealed that the interstitial and intermediate subnuclei of the NTS in rats innervate the pharyngeal muscles, which can trigger the PS [19–21]. Blockade of and lesions to the interstitial and intermediate subnuclei in rats inhibit the PS [22–24]. These findings suggest that the interstitial and intermediate subnuclei may be the key premotor subnuclei receiving sensory information. Moreover, the vagus nerve descends motor commands from the nucleus ambiguous (NA) to the target pharyngolaryngeal regions. Further analyses in cats have also shown that the PS is accompanied by excitation of motor neurons within the dorsal motor nucleus of the vagus nerve and the dorsal part of the NA in cats [18].

**Fig. 1:**
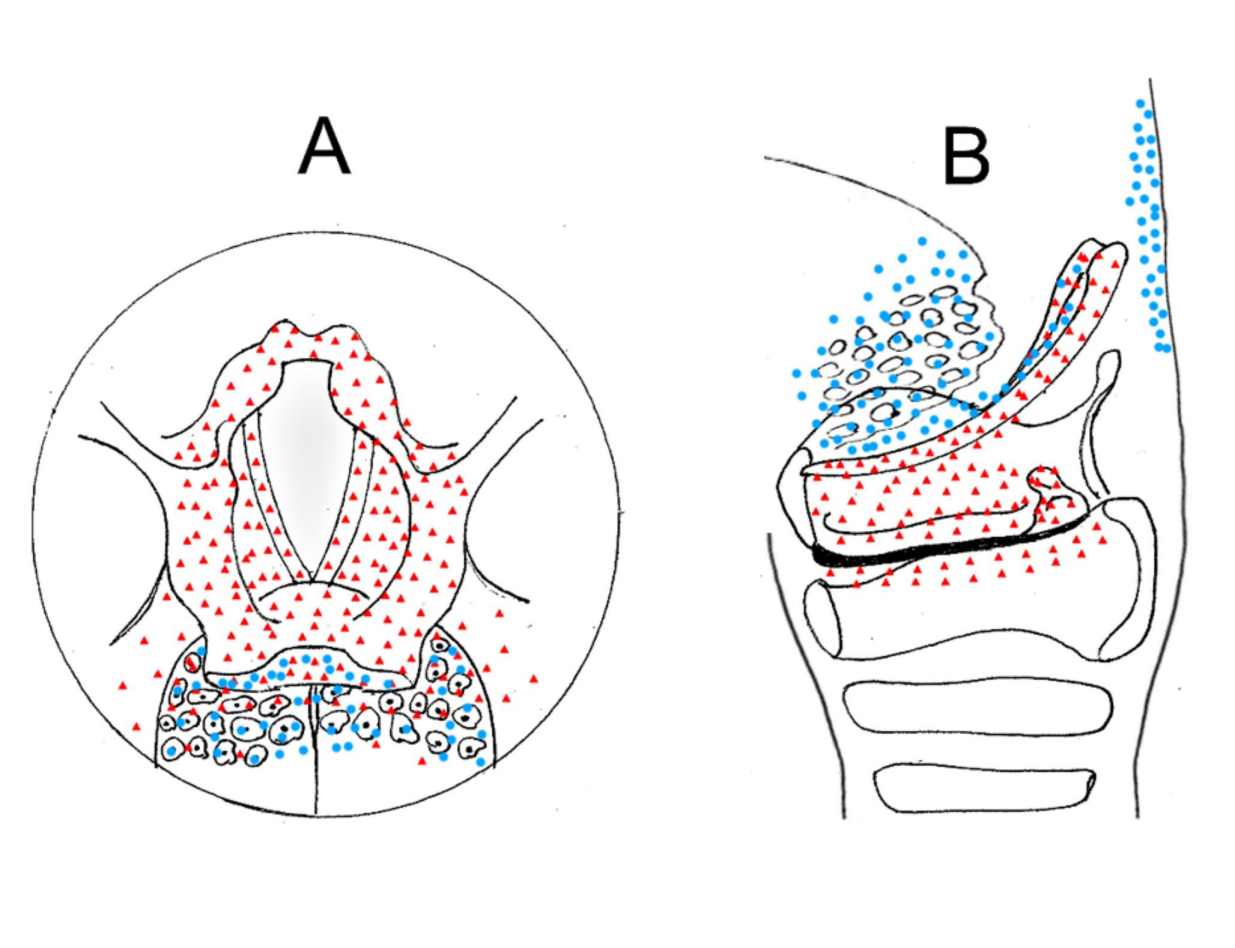
Innervation of pharyngolaryngeal regions by the glossopharyngeal and vagus nerves. This figure shows where pharyngolaryngeal regions are innervated by the glossopharyngeal (blue circles) and vagus nerves (red triangles). **A** and **B**, respectively, indicate superior and lateral views of the pharyngolaryngeal area. This figure is made according to evidence found by Yoshida et al. (2000) [34] and Prescott et al. (2020) [25]

Promisingly, recently conducted research has identified specific vagal sensory neurons, which can play a critical part in triggering a set of motor responses, such as the PS. In this regard, Prescott et al. have mapped the sensory input that these neurons receive to trigger laryngeal defensive responses in the PS in mice [25]. They discovered a part of the vagus nerve, specifically vagal *P2RY1* neurons, controls the PS in addition to a range of common airway defensive responses, including transient apnea, expiratory reflexes, and vocal fold adduction [25–27]. In that study, they revealed that the laryngeal epithelial cells can identify the presence of chemosensory stimulations, specifically by water or acid, in the larynx and convey this information to vagal P2RY1 neurons by adenosine triphosphate neurotransmission. The majority of these neurons innervate laryngeal taste buds, which are mostly placed on the surface of the epiglottis, aryepiglottic folds, vocal folds (both up and down sides), and arytenoid cartilages, and some of the P2RY1 neurons terminate in oropharynx taste buds (see red triangles in Fig. 1). These specific chemosensory stimulations can activate these neurons and immediately trigger a set of defensive motor responses to safeguard the upper airway. This chemosensory-induced motor program leads to transient apnea, vocal fold adduction, and expiratory reflexes in addition to the PS.

Along with the vagus nerve, the glossopharyngeal nerve also plays a significant role in innervating the pharynx. Research has indicated that stimulating the posterior pharyngeal pillars and the posterior pharyngeal wall can induce the PS in cats [28, 29]. Further investigation has shown that these regions are densely innervated by the glossopharyngeal nerve and the pharyngeal branch of the vagus nerve in a human [30]. Studies using different techniques such as degeneration, autoradiography, tracer technique, etc. have demonstrated that the glossopharyngeal nerve sends sensory information to the NTS [31–33]. Yoshida et al. further explained that the glossopharyngeal sensory nerve in cats innervates the nasopharynx, the posterior area of the soft palate, the tongue base, the vallecula, and the lingual side of the epiglottis (see blue circles in Fig. 1) [34]. While several researchers have identified this nerve as the main trigger for the PS [35, 36], this notion might not find support as investigations into the vagus nerve may offer a different perspective. In this regard, several animal investigations have been conducted to determine the primary trigger of the PS [37–42], but the results have not yielded consistent evidence yet [43]. Hence, both the glossopharyngeal nerve (specifically its pharyngeal branch) and the vagus nerve (particularly its pharyngeal and laryngeal branches) can be considered as primary nerves for regulating the PS. Taking together, these pieces of evidence support the traditional perspective on the PS, considering it an exclusive reflex (see Table 1) or non-goal-directed motor response (see Table 1).

Evidence has also shown that the NTS in rats also extensively projects to the dorsal motor vagal neurons (DMV) [44, 45]. Accordingly, observations of mice and cats have shown that the DMV can also participate in the PS [18, 46]. Interestingly, the DMV neurons triggered during the PS are considerably smaller than the ones stimulated during the esophageal phase [18, 46]. The smaller neurons of the DMV are interneurons while the larger ones are motor neurons [47]. Thus, it implies that the smaller DMV neurons may play an inhibitory role (rather than an excitatory role) in the PS, as interneurons are frequently GABAergic and play an inhibitory role. The observed inhibitory effect suggests an active regulatory mechanism aimed at controlling and halting respiration (swallowing apnea) during the PS. In this regard, evidence has revealed that the sensory input can also induce inhibitory effects on respiration through central neural connections in mice [27]. Furthermore, other evidence demonstrated strong deglutitive inhibition of all esophageal neurons, suppressing esophageal peristalsis during the pharyngeal phase of swallowing [17, 48, 49]. Another evidence has indicated that the dorsal motor nucleus in ferrets can modulate the crural diaphragm and lower esophageal activity [50]. This suggests hierarchical control of neural inhibition indicating that distal areas of the alimentary canal are inhibited while the PS is happening. Indeed, the interneurons activities during the PS can control the diaphragmatic inhibitory activities during the pharyngeal phase of swallowing in different contexts [51]. Therefore, it can be assumed that the entire motor sequence of the PS can be modulated through various sensory feedback [17], which means that it may not be entirely reflexive. Accordingly, the pivotal questions now arise: How can the PS process be modulated in response to varying contexts? And which structure(s) may become involved in regulating the PS process to adapt it to contextual demand, e.g., the bolus’s size, texture, etc [17]. ?

**Table 1:** Definition of terms

Central pattern generator	A central pattern generator is a collection of connected neurons that produce a patterned rhythmic behavior following stimulation [52].
Cognitive processing	This term is used in this paper to describe the mental process of perceiving sensory information and comprehending it through thoughts or past experiences.
Efferent copy	An efferent copy is a neural representation of a motor command that has been generated.
Goal-directed pharyngeal swallowing	This term is applied in this paper to describe voluntary responses within the PS, which can be tuned properly based on cognitive input, learned knowledge, and predictions.
Non-goal-directed pharyngeal swallowing	This term is applied in this paper to describe involuntary responses during the PS produced only by the brainstem (not higher brain regions), which do not need cognitive processing. This term can be considered equal to a reflexive PS.

## Goal-Directed Neural Control of the Pharyngeal Phase of Swallowing

Central pattern generators are usually independent entities but can be modulated by various sensory feedback and descending regulations [53, 54]. Thus, there might be some other regions in the brain that intentionally adjust the activity of a central pattern generator whenever needed. This purposeful adjustment occurs to ensure a highly coordinated and accurate outcome. Indeed, there might be one or more centers in the brain to adaptively provide some regulatory commands to support the SCPG by adaptively modulating the process of the PS according to varying contexts.

The cerebellum can provide a working model of an adaptive filter [55–57]. The cerebellum is an essential part of the brain involved in different behaviors, such as motor regulation. It approximately contains 70% of the neurons in the human brain [58], highlighting the complex essence of this vital structure. The proposed effects of the cerebellum on central pattern generation can also suggest a potential connection between the cerebellum and the SCPG [15, 53, 59, 60]. Indeed, the cerebellum may work as a compensatory center with the SCPG to adjust and stabilize swallowing behavior in different contexts (i.e., bolus features, head positions, etc.) being experienced. One analogous example is the vestibulo-ocular reflex [57]. This is an important reflex that is produced by the spinal locomotor central pattern generator (SLMCPG). This reflex stabilizes our gaze to stay balanced and steady while we are moving and experiencing continuous head movements [61–63]. Moreover, the cerebellum constantly receives efferent copies of the ongoing motor commands to the SLMCPG and, thus, may directly participate in producing the required corrections to adjust the reflex if it occurs in an inefficient manner relevant to context [53, 54]. Although the cerebellum is no longer involved in this action when the reflex is correctly adjusted, it has the ability to support learning, and associated neural plasticity, from previous copies of errors to accurately repurpose future patterns [54, 64].

**Fig. 2:**
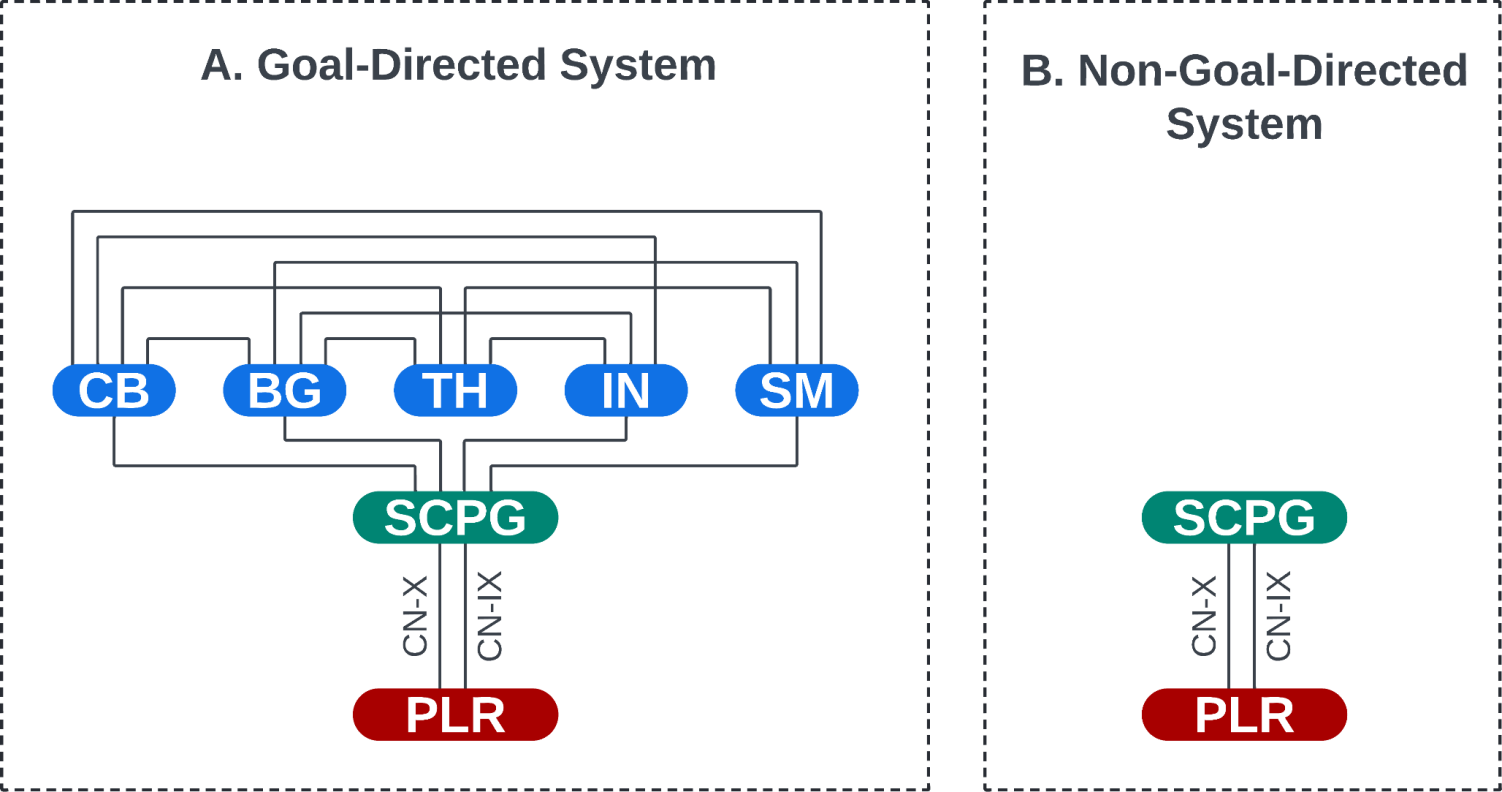
Neural controlling systems of the pharyngeal phase of swallowing. This model illustrates the two neural systems of controlling the pharyngeal phase of swallowing. **(A)** In the goal-directed system, the cerebellum (CB), basal ganglia (BG), thalamus (TH), insula (IN), and the sensorimotor cortices (SM) can directly or indirectly influence the swallowing central pattern generator (SCPG) and regulate the pharyngolaryngeal regions (PLR) involved in the pharyngeal phase of swallowing using the glossopharyngeal and vagus nerves. **(B)** In the non-goal-directed system, however, the swallowing central pattern generator can produce immediate non-voluntary pharyngolaryngeal responses. These responses can mostly result in upper-airway defensive responses rather than an efficient pharyngeal phase of swallowing. **Note**: Various sensory information and cognitive input may enhance the networks between these brain regions and improve the precision and effectiveness of the pharyngeal phase of swallowing within the goal-directed system. Conversely, a reduced perception of sensory and cognitive information may increase the risk of inadequate or inefficient pharyngolaryngeal responses within the pharyngeal phase of swallowing. If pharyngeal swallowing proceeds in an insufficient manner, the cerebellum, basal ganglia, insula, and the sensorimotor cortices can be involved and bind various sensory-motor information to modulate (calibrate) the activities of the swallowing central pattern generator until it produces appropriate responses according to the current context. In addition, these brain regions, especially the cerebellum, can enable individuals to learn the appropriate responses and then form appropriate predictive patterns for future contexts. This capability can allow one to provide goal-directed pharyngolaryngeal responses before the PS occurs. This flowchart was made by Lucidchart

The connection between the SLMCPG and the cerebellum might also suggest a similar relationship between the cerebellum and SCPG (see CB in Fig. 2). Accordingly, the SCPG can independently act to produce swallowing in response to the sensory stimulation of the pharyngolaryngeal areas, but it also sends a copy of sensory feedback to the cerebellum (see CB in Fig. 2) either through the olivocerebellar fibers, mossy fibers, or reticular formation [65, 66]. In this regard, limited evidence predominantly points to olivocerebellar fibers with the inferior olivary nucleus as a key player in motor learning and motor timing [67], and the scarce evidence available suggests a potential correlation between inferior olivary malformation and abnormal involuntary pharyngolaryngeal rhythmic movements [68, 69]. Whichever that pathway may be, the cerebellum can likely learn from comparing sensory feedback and the efferent copies. This can allow the cerebellum to support the SCPG as soon as a motor response encounters a breakdown. The cerebellum may also modulate the SCPG’s future activities according to expectations based on what it has learned so far. If the cerebellum can predictively tune the swallowing reflex according to what it has learned before, the PS can therefore have the capability to function as a form of goal-directed behavior (see Table 1) [70, 71] to intentionally match the motor response to the task at hand [72]. This concept supports the idea that individuals can achieve efficient swallowing behavior when they are aware of what they are about to swallow. In this regard, Leopold et al. proposed a pre-oral phase for the swallowing mechanism at which orientation about the meal (i.e., bolus characteristics) assists the following swallowing phases before swallowing starts [73]. As a result, sensory and cognitive cues related to the meal can play a crucial role in shaping our pharyngolaryngeal motor behavior during swallowing [74]. Thus, this can support the notion that the activity of these hierarchical structures may take place within the state of cognitive processing (see Table 1) of perceived information likely derived from different sensory modalities, although the exact extent of participation from each modality remains unclear.

Behavioral evidence has indicated that rats can swallow water while in the process of licking without interrupting their licking behavior [75]. However, the absence of cerebellar output leads to a notable slowing of the licking rhythm, amounting to a reduction of 15–19% [76–79]. This implies that the cerebellum may play a role in modulating temporal tuning in the PS [80]. Emerging evidence has further shown that compromised cerebellar regions can cause pharyngeal dysphagia. In this regard, Huang et al. (2023) found that 11.45% of people with cerebellar infarct experienced dysphagia [81]. They also discovered that older people were more susceptible to dysphagia following the infarction. In addition, Hajipour et al. (2023) investigated dysphagia among people with cerebellar stroke [82]. They also found similar results, although the rate of prevalence for dysphagia was 52.9% among those people. They discovered a significant connection between dysphagia and aspiration. Interestingly, they further observed that the gag reflex faded away in almost all individuals. Moreover, it has been observed that transcranial magnetic stimulation of the cerebellar vermis can lead to pharyngeal motor responses in humans [83]. Taken together, these results can account for the role of the cerebellum in the PS (see CB in Fig. 2) [84].

Alongside the cerebellum, other brain regions that are partially lateralized in the right hemisphere can also play a role in the synergy of the PS and reciprocate information with the cerebellum or each other. These regions could also be associated with the cognitive processing of perceived information, which is capable of voluntarily triggering or controlling the PS process (a dry swallow can be a noteworthy example wherein one intentionally produces a pharyngeal response without an actual bolus). The following paragraphs discuss evidence regarding the possible role of the basal ganglia, thalamus, insula, and sensorimotor cortices in controlling the PS process.

The basal ganglia are a set of interconnected subcortical nuclei comprised of the striatum, globus pallidus, and subthalamic nucleus. Damage to the basal ganglia reduces its output specificity and topographical precision according to a particular goal, which can disrupt its main role of regarding accurate and on-time motor output [85]. In this regard, Rodriguez-Oroz et al. (2001) described a subset of neurons within the globus pallidus that particularly respond to motions across orofacial regions [86]. Neuroimaging investigations of swallowing have shown that the complex process of swallowing necessitates the engagement of several brain regions, including components of the basal ganglia, specifically the internal segment of the globus pallidus [87]. Insufficient laryngeal elevation and glottal closure during the pharyngeal phase of swallowing have both been recently reported to be linked to damage to the external capsule, of the globus pallidus [88]. Moreover, lesions to pallidothalamic fibers can be associated with weakened anterior hyoid excursion [88]. This evidence highlights the role of the basal ganglia, especially the globus pallidus, together with the thalamus and the pallidothalamic fibers in regulating the movements during the pharyngeal phase. Therefore, this evidence suggests that disruptions and dysfunctions of basal ganglia can interfere with the intricate sensorimotor neural pathways and their integrated coordination, which is essential for safe and efficient swallowing [89, 90].

The basal ganglia has been recently suggested to play a role in novel motor goal determination, assisting the cerebellum in reducing residual motor response errors in such situations [59]. This capability of the basal ganglia also enables it to modulate central pattern generators in the brainstem, especially in childhood [59]. This reciprocatory relationship between the basal ganglia and the cerebellum can minimize the error while one is about to produce a novel motor response. Taken together, the basal ganglia (see BG in Fig. 2) and the cerebellum (see CB in Fig. 2) may support individuals when they are about to encounter a novel context.

The thalamus is composed of nuclei that play roles in sensory processing through connections with other cortical and subcortical regions, especially sensory areas [91, 92]. The thalamus also functions as a sensory integration hub because it transmits different sensory information regarding gustation and deglutition to cortical and subcortical loci that are part of the basal ganglia-thalamo-cortical loops [93]. Moreover, thalamic activation has been observed during tongue movements [94], coughing [95], and throat clearing [96].

Bieger and Hockman (1976) demonstrated that applying electrical stimulation to the SLN, in conjunction with areas of the diencephalon beyond the corticobulbar pathway, can enhance the process of the PS in cats [97]. A recent review has also provided further empirical evidence following structural damage in the *right* thalamus as well as functional abnormalities in the *left* thalamus [98]. Qin et al. (2023), pointed out that both structural lesions and functional irregularities within thalamic regions were associated with swallowing disorders [98, 99], including delayed the PS [100]. Wilmskoetter et al. (2019) also revealed a significant correlation between damage to thalamic nuclei and deficient anterior excursion of the hyoid bone during swallowing. This piece of evidence suggests that the thalamus may also have an integral role in enabling effective anterior hyoid motion due to its anatomical connections with structures involved in swallowing coordination such as the striatum’s basolateral nuclei, pons, and medulla oblongata [88]. Interestingly, Lapa and colleagues have also demonstrated that deep brain stimulation of the thalamus (the ventral intermediate nucleus) can cause pharyngeal dysphagia (premature bolus spillage and impaired glottal closure) and result in laryngeal penetration and aspiration before swallowing [101]. This evidence highlights the prominent sensory processing role of the thalamus in the coordination, timing, arrangement, and sequencing of purposeful movements during the PS process (see TH in Fig. 2).

The insula is a cortical region situated between the frontal and temporal lobes, deeply within the Sylvian fissure of the brain. Evidence has identified the insula as the primary gustatory and vibrotactile cortex [102]. The insula has connections to various subcortical, cerebellar, and brainstem regions, which are considered to be involved in the PS (see IN in Fig. 2) [103]. Im et al. (2018) reported that insular lesions caused delayed pharyngeal transition in people with subcortical strokes [104]. Additionally, a meta-analysis of literature has recently revealed that the insular cortex engages in pharyngeal transition and its damage can result in compromised swallowing initiation, delayed pharyngeal transition, reduced laryngeal elevation, insufficient vocal fold closure, and aspiration [105]. Lowell et al. (2012) have further described the insula as an essential region engaged in voluntary swallowing [106], which has been confirmed by further evidence [88, 107]. This information suggests that the insula may play a role in coordinating sensory and motor information, particularly during voluntary PS, with the help of other brain regions to facilitate on-time and smooth movements of the PS components (see IN in Fig. 2). Therefore, its damage may lead to compromised PS process and aspiration [105].

In addition to the insula, evidence has also suggested that the sensorimotor cortices can also be involved in the PS process [108]. In this regard, transcranial magnetic stimulation (TMS) of the anterolateral primary motor cortex can be related to neuromuscular activation of the pharyngeal region [109, 110]. Mistry et al. conducted a study in 2007 to examine the effect of the primary motor cortex inhibition on the PS reaction time by measuring the neuromuscular activity of the pharynx [111]. Hence, they asked their participants to produce swallowing responses after receiving electrical cues. Interestingly, they found that the primary motor cortex inhibition can cause inhibitory signals toward the SCPG and modulate the initiation of the PS [111]. This piece of evidence can suggest that the primary motor cortex can be involved in the voluntary initiation of the PS [112]. Moreover, electrical stimulation of the pharyngeal area [113] or air-pulse stimulation of the laryngeal region [114–116] can increase activation of the primary somatosensory cortex. On the other side, pharyngeal anesthesia can reduce activity in the primary somatosensory cortex [117]. This cortical region has been suggested to be involved in processing sensory information (during swallowing) and regulating the SCPG [116, 118, 119]. Evidence has further shown that damage to the primary motor and somatosensory cortices can also compromise the PS [108], causing pharyngeal delay, pharyngeal residue, compromised laryngeal closure, and aspiration [120, 121]. In these cases, the SCPG may still have the capacity to generate the PS even though these cortical regions are compromised, but the produced responses would be less coordinated [119]. These pieces of evidence can support the idea that the sensorimotor cortices can be involved in voluntary initiation or regulation of the PS as well as processing sensory information during swallowing the PS process (see SM in Fig. 2).

Finally, the brain regions can directly or indirectly communicate information with the SCPG through the motor pathways to influence the PS. These pathways may include both pyramidal and extrapyramidal tracts [122–124], but the precise extent to which each pathway and its subdivision contribute to the PS process still remains unclear. In general, the cerebellum is extensively connected to the brainstem and the thalamus [125, 126]. The olivocerebellar fibers, mossy fibers, or reticular formations can mediate neural communication between the SCPG and the cerebellum [125, 127]. In addition, the cerebellum can facilitate coordination of neural communication among other cortical regions through the cerebello-thalamo-cortical pathway [128]. The basal ganglia can also communicate information with the SCPG through a neural network [129]. In addition, the cortico-basal ganglia-cerebellar network allows the basal ganglia to reciprocate information with the cerebellum and cortical regions [130]. The thalamus may not directly communicate with the SCPG, but it can be considered a primary sensory coordinator between the cerebellum, basal ganglia, and cortical regions [131]. The thalamus can also reciprocate somatosensory information with the insula [132]. Deng et al. have also demonstrated that the insula projects to the NTS (sensory nuclei of the SCPG) through the pyramidal tract neurons [133]. Moreover, the insula may also have a complex network with the basal ganglia, which may be responsible for the detection, recognition, and expression of disgust [134]. The insula may also reciprocate interoceptive information with the cerebellum to support interoception system in humans [135]. Corticofugal fibers may also mediate information between the cortical sensorimotor areas and the SCPG [136]. Figure 2 displays the networks between these regions.

Hemispheric lateralization has been indicated in the control of swallowing in the vast majority of humans, but the dominant activation side appears to vary among individuals [137]. The majority of evidence has shown that lesions in the right hemisphere led to a greater severity of the PS impairments [120, 121, 138–143]. For example, lesions in the right hemisphere can cause compromised pharyngeal contraction, pharyngeal residue, laryngeal elevation insufficiency, and pharyngeal delay, all of which significantly increased the risk of aspiration. Accordingly, neuroimaging studies have also shown left and bilateral hemispheric engagement during the oral preparatory phase of swallowing. They indicated that a strong shift to right hemispheric activation occurs as the swallowing process progresses in healthy and post-recovery stroke individuals [144–146]. Notably, Holtmann et al. also observed that lesions of the insula-basal ganglia network in the left hemisphere can decrease the sense of disgust, while lesions of this network in the right hemisphere can increase the sense of disgust in humans [134]. Interestingly, Bai et al. have further found that the improvement of causal effect strength from the cerebellum to the insula can reduce the sense of disgust [135]. These pieces of evidence suggests that the insula-basal ganglia network in the right hemisphere may also play a role in inhibiting disgust, potentially aiming to suppress associated outcomes such as nausea or vomiting when encountering familiar, experienced, or non-invasive substances (boluses) with the interoceptive information received from the cerebellum even before the PS occurs. This kind of goal-directed and survival-oriented behavior are vitally required for reducing a risk of getting poisoned or facing life-threatening consequences. Thus, the cumulative evidence suggests that the control of the PS may be partially lateralized to the right cerebral hemisphere.

Figure 2, Key Figure, shows both neural controlling systems of the PS based on the evidence discussed in this paper.

## Clinical Translation

The present review highlighted that the PS can be regulated through two systems: goal-directed and non-goal-directed. As mentioned earlier, this type of regulation may not be entirely categorized as either goal-directed or non-goal-directed, but rather falls along a spectrum between these two controlling systems, which can change according to the current context and task at hand. Furthermore, many of the brain regions and connecting pathways discussed here may be located close to each other. This can make them susceptible to compromise from various types and severities of neurological diseases or disorders. Hence, various neurological conditions may cause a combination of different signs and symptoms. Although the subsequent clinical translation should be approached with caution, gaining a broad perspective on the signs and symptoms of compromised goal-directed or non-goal-directed systems of the PS might aid clinicians and researchers in a deeper understanding of swallowing neurophysiology.

According to the findings of this review, damage to the SCPG can generally cause a lack of PS. Injuries of the glossopharyngeal can lead to severely impaired PS, weakened sensory perception (particularly in the nasopharynx, the posterior area of the soft palate, the tongue base, the vallecula, and the lingual side of the epiglottis), weakened pharyngeal motor responses, increased risk of aspiration. Also, issues with the vagus nerve can cause severely impaired PS, attenuated sensory perception (especially on the surface of the epiglottis, aryepiglottic folds, vocal folds, and arytenoid cartilages, and to some extent, oropharynx taste buds), laryngeal weakened motor responses, compromised upper-airway defensive responses (such as inappropriate transient apnea, vocal fold adduction, and expiratory reflexes), and greater risk of aspiration. Dysfunction or damage to the SCPG, glossopharyngeal, or vagus nerve can increase the risk of aspiration, particularly silent aspiration. Cerebellar issues may lead to flawed adaptability to various contexts (stimuli), temporal incoordination among PS components, delayed movements in PS components, poor gag reflex, and aspiration. Basal ganglia complications can cause sensorimotor errors/delays in PS components and aspiration, especially in unfamiliar contexts. Thalamic issues can result in atypical somatosensory perception, delayed or uncoordinated function of PS components, and aspiration. Insula problems can produce an unusual sense of disgust, poor voluntary initiation of the PS, and aspiration. Damage to sensorimotor cortices can lead to weakened voluntary initiation of the PS and incoordination of the PS mechanism.

These signs and symptoms may also arise if the connections between these regions are compromised. Damage or dysfunction of the olivocerebellar fibers, mossy fibers, or reticular formations can cause PS signs similar to cerebellar dysfunction. Issues in the basal ganglia-cerebellar network may cause impaired adaptability to different contexts, sensorimotor errors in PS components, temporal incoordination among PS components, delayed movements in PS components, compromised gag reflex, and aspiration. Basal ganglia-SCPG network problems may lead to sensorimotor errors in the PS, especially in novel contexts. Compromised thalamic networks may negatively impact somatosensory perception, the coordination of PS components, the functions of other brain regions, and the risk of aspiration. Problems with the connection between the insula and SCPG may lead to a compromised sense of disgust, impaired voluntary initiation of the PS, and aspiration. Issues with the connection between the insula and basal ganglia can result in a compromised sense of disgust, impaired voluntary initiation of the PS, and sensorimotor errors or delays, particularly in novel contexts. They may also increase the possibility of aspiration. Injury or malfunction of the insula-cerebellum link may cause an uncommon sense of disgust, poor voluntary initiation of the PS, weak flexibility of the PS within various contexts, uncoordinated or delayed timing of PS mechanism, compromised gag reflex, and an increased risk of aspiration. Finally, issues with links between sensorimotor cortices and the SCPG may lead to poor voluntary initiation of the PS and incoordination of the PS components.

## Concluding Remarks and Future Perspectives

In conclusion, the results of this study support the idea that the PS can be controlled through two controlling systems, goal-directed and a non-goal-directed. This implies that its nature may not be exclusively reflexive or voluntary but may be a combination of both that are affected by the cognitive processing of perceived information. Accordingly, emerging evidence has also demonstrated the influence of cognitive processing on the PS in neurotypical individuals and people with neurological issues [74, 147, 148]. The discussion also highlighted the importance of the pre-oral phase in addition to the more widely recognized classification of swallowing phases (oral, pharyngeal, and esophageal). Moreover, it showed how the pre-oral phase (events) can affect the PS process. While these findings are promising, the next step should be to understand how and to what extent these two neural systems may collaborate to control the PS process in different human populations within different contexts.

Although the precise signs and symptoms of issues related to these controlling systems may still remain unclear, these impairments can typically lead to aspiration and place the affected individual at risk of serious consequences. Therefore, future research should focus on exploring the capabilities of these two neural controlling systems of the PS and their precise pathologies since the PS may become compromised under different neurological conditions, such as stroke [149], multiple sclerosis [150], Parkinson disease [151], etc. This or future evidence may potentially challenge our traditional understanding of the PS as an exclusive reflexive or voluntary behavior. However, it can contribute to better support for individuals with dysphagia, particularly those with cognitive impairments and neurological issues.

## Data Availability

The authors verify that the data supporting the findings of this review are available within the article.
